# Improving tuberculosis contact investigation in Chhattisgarh, India: early findings from operational research

**DOI:** 10.5588/pha.26.0014

**Published:** 2026-08-24

**Authors:** C. Malik, V. Gupta, K. Shringarpure, H.A. Gupte, Y. Kalkonde, Y. Jain, S. Khanna, M. Damani, N.N. Shah, K. Sonwani, V.R. Keshri, H.D. Shewade

**Affiliations:** 1 Sangwari-People’s Association for Equity and Health, Surguja, Chhattisgarh, India;; 2 State Health Resource Centre, Raipur, Chhattisgarh, India;; 3 Department of Community Medicine, Medical College Baroda, Vadodara, Gujarat, India;; 4 Narotam Sekhsaria Foundation, Mumbai, Maharashtra, India;; 5 Department of Health and Family Welfare, National Tuberculosis Elimination Programme (NTEP), National Health Mission, Raipur, Chhattisgarh, India;; 6 Department of Health and Family Welfare, Directorate of Health Services, Raipur, Chhattisgarh, India;; 7 Jindal School of Public Health and Human Development, O. P. Jindal Global University, Sonipat, India;; 8 Division of Health Systems Research, ICMR-National Institute of Epidemiology, Chennai, India.

**Keywords:** tuberculosis, primary health care, TPT, decentralisation, HCI, M-PHISOR

## Abstract

**SETTINGS:**

Chhattisgarh state, central India, implemented measures to improve household contact investigation (HCI) monitoring and decentralising HCI to nearest primary care facility.

**OBJECTIVES:**

Comparing bacteriologically confirmed adults (≥18 years) with tuberculosis (TB) from October to December in 2023 and 2025, to assess changes in undergoing HCI, TB diagnosis, and TB preventive therapy (TPT) initiation (overall and children [<5 years]) after introducing HCI monitoring indicators and decentralising HCI to primary care.

**DESIGN:**

Operational research using cross-sectional descriptive analysis of programme data.

**RESULTS:**

Districts (n = 33) reporting monthly HCI indicators increased from 2 (6%) in October 2025 to 20 (60%) in January 2026. Notifications dropped from 4,221 to 3,196 and diagnosis through sputum microscopy from 1,315 to 51; while diagnosis by rapid molecular tests increased from 2,290 to 2,774. Marginal improvements occurred in current treatment facility as primary peripheral health institutes (46%–54%), undergoing HCI (75%–82.5%), average household contact identified per person with TB (2.8–3.1), and household contacts initiated on TPT (64%–70%). These changes were not observed among child (<5 years) contacts. TB diagnosis among contacts did not improve (overall and children [<5 years]).

**CONCLUSION:**

Early findings for HCI monitoring are encouraging. However, prioritising child (<5 years) contacts and bacteriological confirmation at primary care level is urgently needed.

Tuberculosis (TB) is the leading cause of death caused by an infectious disease. The global gap between the annual estimated incidence and notifications is 2.4 million.^1^ The global reduction in TB incidence rate between 2015 and 2023 was 12%, well below the WHO END TB strategy goal of 50% reduction by 2025.^1^ To reduce TB incidence, contact investigation for those living with a person with pulmonary TB, and TB preventive therapy (TPT), is one of the components of the END TB strategy.^2^ Household contact is a person who shares the same enclosed living space for one or more nights or for frequent or extended daytime periods during the 3 months to initiation of current TB treatment.^3^ TPT, as a health care intervention, helps reduce the risk of progression from TB infection to TB disease. Several studies globally and in India have shown that household contact investigation (HCI) helps to improve TB disease detection and TPT coverage.^4–9^ The estimated global coverage of TPT initiation among household contacts in 2023 was 25%.^1^ India launched and expanded TPT coverage under the National Tuberculosis Elimination Programme (NTEP) to all contacts in 2021.^3^ Against the global and national targets of 90% for all age groups, TPT initiation among eligible household contacts in India in 2023 was 27% across all age groups and 60% among children (<5 years).^10^

The Government of India is strengthening the primary care facilities by upgrading primary health and sub-health centres (which serve populations of 30,000 and 5,000, respectively) to health and wellness centres (HWCs) to offer a comprehensive package of primary care services closer to the community. It mandates decentralised TB services at HWCs, including HCI, presumptive TB case detection, and TPT initiation and follow-up.^11^ Our statewide operational research in Chhattisgarh state (central India) looked at the status of HCI in the state for all those notified with pulmonary TB during October–December 2023.^12^ The findings re-iterated the need to prioritise HCI in the NTEP, decentralising it to the primary care level, and regularly monitor it in the programme. A monitoring indicator slide for HCI was suggested (https://figshare.com/s/09837c44766a32559e5a).^12^ The findings were shared with state-level officials, and adopted to be used by all the districts of the state. The recommendations were issued as an order to all districts. Online training to support the implementation for district-level officials was done. District officials were supported to functionalise HCI at the primary care level and submit the HCI indicator slide monthly.

The present operational research documented early implementation findings to decentralise HCI after introduction of monthly indicator slide. The specific objectives were to study among those notified with pulmonary TB, if this implementation improved HCI coverage, TB detection, and TPT initiation for all ages and children < 5 years in October–December 2025 when compared to October–December 2023. The proportion of pulmonary TB overall and those being treated at primary care level was also compared.^12^

## METHODS

This operational research was conducted using cross-sectional descriptive analysis involving routinely captured secondary data under routine programme settings. We have compared across two time periods: October–December 2023 and 2025 for before–after comparison.

### Settings

Chhattisgarh has 3,500 HWCs at the primary care level, out of which 778 (mostly at primary health centres) have designated microscopy centres for diagnosing TB using microscopy, 150 community health centres that function as TB units at the secondary care level where nucleic acid–based amplification tests (NAAT) and X-rays are available, and 33 district hospitals and 11 medical colleges at the tertiary level in the public health system. All health facilities where a medical officer can diagnose and treat TB are referred to as peripheral health institutes (PHIs). HWCs, where a community health officer and nurse are stationed as health care workers, are also included as PHIs in the programme.^10^ The diagnosing health facility is where TB is diagnosed; and the current health facility is where treatment for TB, once started at the diagnosing health facility, is continued. They could be the same for a person, or if needed the current facility can be changed to the PHI nearest to the person.

The programme staff in the public health sector conduct an HCI for all persons with pulmonary TB, including those notified in the private sector at the current PHIs. Household contacts with symptoms of TB (irrespective of age) are referred to PHIs for confirmation of diagnosis for TB. Children (<5 years) are to be started on TPT after ruling out TB, and those >5 years are to be put on TPT after testing for TB infection. This guidance to expand TPT for age > 5 years was issued in 2021.^3^

### Implementation of recommendations

Based on the recommendations of previous operational research,^12^ the state implemented: 1) functionalising decentralisation of HCI by shifting the treatment of people with TB to their nearest primary care PHI and 2) reporting of monthly HCI monitoring indicators on the 15th of every month. District-level programme managers and senior treatment supervisors (who are stationed at the community health centres and are supposed to perform HCI) were trained to make the HCI monitoring indicators slide for their respective districts. The training also focused on the importance, process, and follow-up of HCI to be done at the primary care PHI level for those notified with pulmonary TB. The number of districts that were able to generate the monthly indicator of HCI were tracked using Excel format from October 2025 onwards (Figure). Follow-up meetings with district- and state-level officials were done to monitor the progress. We shall continue to monitor quarterly changes in HCI along with regular refresher trainings to document if changes in HCI are attributable to the implementation.

**FIGURE.**
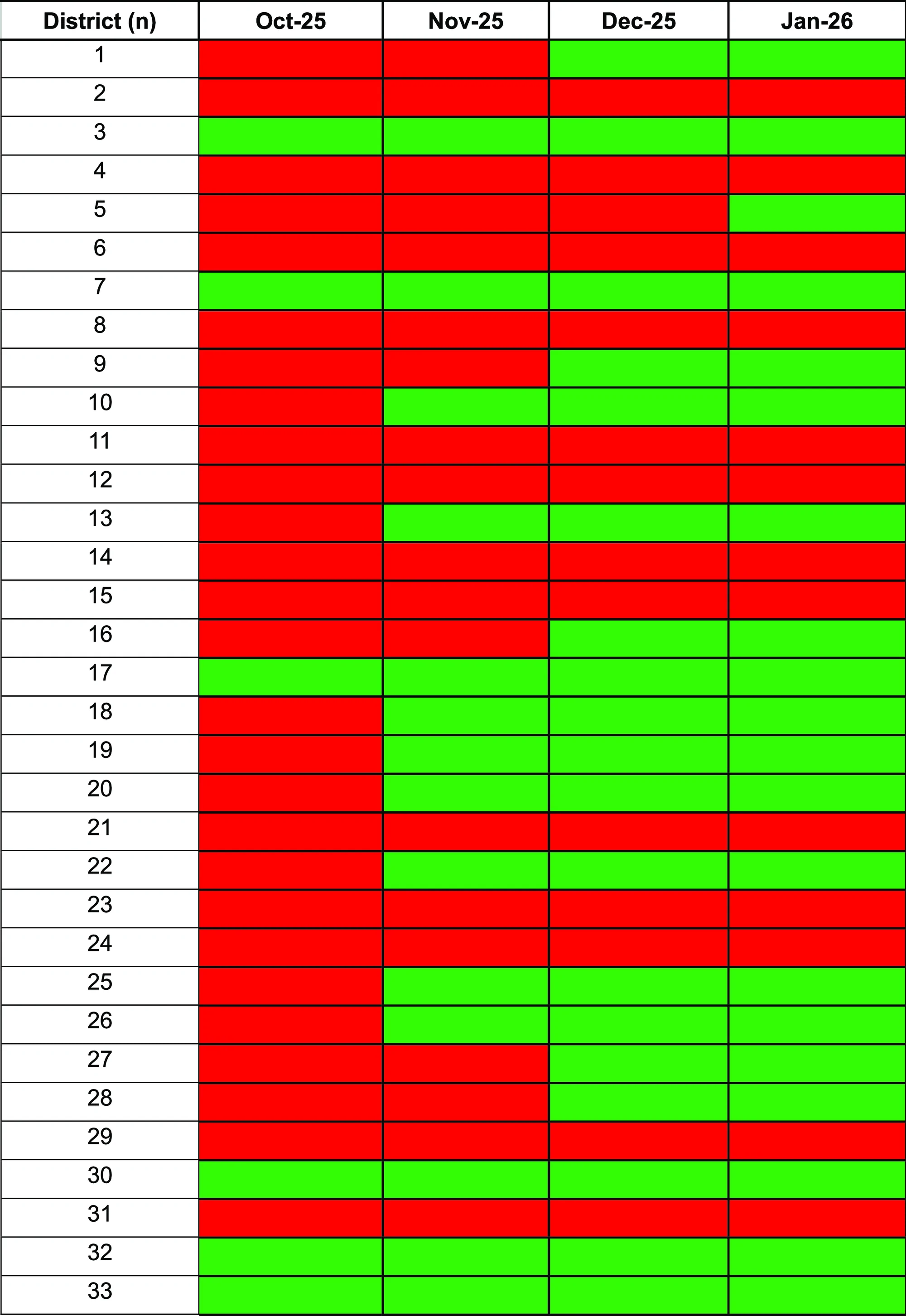
District-wise submission of monthly household contact investigation (HCI) monitoring slide, Chhattisgarh, India, October 2025–January 2026. Districts are represented as numbers from 1 to 33; green = HCI indicator slide submitted; red = HCI indicator slide not submitted.

### Study population

We included all adults (≥18 years) with bacteriologically confirmed pulmonary TB notified during October–December 2025 and October–December 2023 in Chhattisgarh.

### Variables and sources of data

The individual-level socio-demographic, clinical, and health system–related characteristics were extracted from the notification register of *Ni-kshay*,^13^ the web-based case-based TB information management system of NTEP. For each adult with bacteriologically confirmed pulmonary TB, the following aggregate numbers were also extracted from contact tracing register of *Ni-kshay*: household contacts, TB diagnosis, and TPT initiation (overall and among children [<5 years]).

### Data analysis

Data for 2025 cohort were extracted in March 2026, cleaned as per the codebook (https://figshare.com/s/7f3834a17cdb9e872811), and analysed using EpiData Analysis software (v 2.2.2.183 EpiData Association Odense, Denmark). Data were downloaded for those on TB treatment whose current facility was in the state of Chhattisgarh (using the current filter in *Ni-kshay*) in Microsoft Excel format. The two datasets were merged using a unique episode identifier. After merging the databases, if aggregate numbers were not reported in the contact tracing register, it was recorded as not undergoing HCI. Those with PHI at HWCs and primary health centres were labelled ‘primary care facilities’, community health centres were labelled ‘secondary care facilities’, and district hospital and medical colleges were labelled as ‘tertiary care facilities’ in the public sector. Numbers and proportions of those notified with pulmonary TB, HCI done, those with current facility as primary PHI, and those diagnosed with TB and initiated on TPT (along with their clinical, socio-demographic, and health system–related factors) were captured for both October–December 2023 quarter and for October–December 2025 quarter.

### Ethical statement

Ethics approval was obtained from Institutional Human Ethics Committee of ICMR National Institute of Epidemiology (ICMR-NIE), Chennai, India (NIE/IHEC/A/202408-04, dated 11 September 2024 and renewed on 26 June 2025). As this operational research involved analysis of individual and aggregate secondary programme data after removing personal identifiers, we received a waiver for written informed consent from the ethics committee.

## RESULTS

Out of 33 districts in the state, the number of districts submitting monthly indicator reports increased from 2 (6%) in October 2025 to 15 (45%) in December and 20 (60%) in January 2026 (Figure).

Among 3,196 people notified with bacteriologically confirmed pulmonary TB during October–December 2025, 70% were male, 85% were diagnosed using NAAT, and 93% were newly diagnosed with pulmonary TB. 70% were notified in secondary care facilities (70.2%), while 15% were notified from the private sector and 7% from primary care PHIs. Table 1 compares baseline social demographic, clinical, and health system–related factors of index TB notified between October–December 2023 and 2025. There was a significant drop in the number of notifications from 4,221 to 3,196. A major reason for this was a drastic drop in the numbers diagnosis through sputum microscopy (1,315–51) while the number diagnosed by rapid molecular tests marginally increased (2,290–2,774).

**TABLE 1.** Socio-demographic, clinical, and health system–related characteristics of adults (age > 18 years) with bacteriologically confirmed pulmonary TB during October–December 2023 (N = 4,221) and October–December 2025 (N = 3,196) in Chhattisgarh, India.

Patient characteristics		October–December 2023	October–December 2025
		N (%)^A^	N (%)^A^
Total		4,221	3,196
Age			
Age 18–44 years		2,218 (52.5)	1,648 (51.6)
Age 45–59 years		1,113 (26.4)	837 (26.2)
Age > 60 years		890 (21.1)	711 (22.2)
Gender			
Men		2,879 (68.2)	2,240 (70.1)
Women		1,342 (31.8)	956 (29.9)
Others			
District^B^			
Predominantly non-tribal		3,152 (74.7)	2,290 (71.7)
Predominantly tribal		1,069 (25.3)	906 (28.3)
Clinical characteristics			
New		3,440 (81.6)	2,958 (92.6)
Previously treated		390 (9.2)	212 (6.6)
Missing		313 (7.4)	26 (0.8)
Resistant		78 (1.8)	0 (0)
Basis of diagnosis			
ZN staining		1,315 (31.2)	51 (1.6)
CBNAAT/Truenat		2,178 (51.6)	2,728 (85.4)
LPA		112 (2.7)	46 (1.4)
Culture		11 (0.3)	4 (0.1)
Others		605 (14.2)	367 (11.5)
HIV status			
Non-reactive		4,019 (95.3)	3,083 (96.5)
Reactive		61 (1.4)	53 (1.6)
Missing^C^		141 (3.3)	60 (1.9)
DM			
Negative		3,434 (81.3)	2,711 (84.8)
Positive		429 (10.2)	306 (9.6)
Missing/unknown^C^		358 (8.5)	179 (5.6)
Diagnosing health facility			
Primary (HWC/PHC)		453 (10.7)	222 (6.9)
Secondary (CHC/DH)		2,140 (50.8)	2,242 (70.2)
Tertiary (medical college)		382 (9)	257 (8)
Private^D^		864 (20.5)	468 (14.6)
Others^C^		382 (9)	7 (0.2)

TB = tuberculosis; ZN staining = Ziehl–Neelsen staining; CBNAAT = cartridge-based nucleic acid amplification test; Truenat = a rapid molecular test based on polymerase chain reaction technology to detect TB; LPA = line probe assay; Resistant = drug-resistant TB; HIV = human immunodeficiency virus; DM = diabetes mellitus; HWC = health and wellness centre; PHC = primary health centre; CHC = community health centre; DH = district hospital.

A Column percentage.

B District – based on the districts in which the current residence lies; Districts > 50% population belonging to scheduled tribes (‘tribal’) and those with a population less than 50% as ‘non-tribal’.

C Missing – data not available and entered.

D Private – all non-government health facilities.

Between October–December 2023 and 2025, marginal improvements were observed in the current treatment facility as primary PHI (46%–54%), undergoing HCI (75%–83%) – see Table 2 – average household contact identified per person with TB (2.8–3.1), and household contacts initiated on TPT (64%–70%). The number of household contacts screened overall and children (<5 years) was 9,771 and 952, respectively, during October–December 2025. Among them, 96 (0.9%) were diagnosed with TB which was similar (0.9%) to 2023. For children <5 years, there was a decrease in proportion diagnosed with TB (0.7%–0.4%) and those initiated on TPT (73%–61%) – see Table 3.

**TABLE 2.** Pulmonary TB notifications and household contact investigation (HCI) coverage overall and those with current facility as primary care PHI, before and after implementation, October–December 2023 (N = 4,221) and October–December 2025 (N = 3,196).

	October–December 2023	October–December 2025
	N (%)^r^	N (%)^r^
Persons notified with TB	4,221	3,196
Current facility as primary care PHI	1952 (46.2)	1720 (53.8)
Underwent HCI	3,177 (75.3)	2,638 (82.5)

Tb = tuberculosis; PHI = peripheral health institute; r = row percentage; primary care PHI = health and wellness centres/primary health centres.

**TABLE 3.** State-level household contact investigation (HCI) coverage, contacts identified, TB detected, and TPT initiation before and after implementing district-level monthly HCI indicator reporting, October–December 2023 and October–December 2025.

	October–December 2023		October–December 2025	
	All age groups	Children (<5 years)	All age groups	Children (<5 years)
	N (%)	N (%)	N (%)	N (%)
Total notified	4,221		3,196	
Number of HHC investigations done	3,177 (75.2)		2,638 (82.5)	
Total number of HHCs screened	12,071	1,230	9,771	952
Number of people with TB detected through contact tracing^A^	109 (0.9)	9 (0.7)	96 (0.9)	4 (0.4)
Number of contacts for whom TPT initiated^A^	7,740 (64.1)	903 (73.4)	6,834 (69.9)	577 (60.6)

TB = tuberculosis; TPT = TB preventive therapy; HHC = household contact.

A Percentage of HHCs screened.

## DISCUSSION

Our study represents early implementation findings from a statewide effort to improve HCI. Monthly reporting of HCI monitoring indicators was not done by all districts in the state till January 2026. Compared with October–December 2023, there was a marginal improvement in referral to the nearest primary health facility as the current treatment facility, in HCI and TPT initiations. However, these early changes were not reflected among children (<5 years). TB diagnosis among contacts did not improve (overall and among children [<5 years]), one possible reason being the drastic drop in the numbers diagnosed by sputum microscopy (the mainstay of TB diagnosis in primary health centres).

This operational research was done at the state level in a real-world setting; hence, the findings are representative of the state. However, we can’t attribute the improvement only because of the steps taken to strengthen HCI. These are early changes, and long-term follow-up shall be done to document if the changes are sustained and attributable to what has been planned. Despite these limitations, there are some relevant policy- and practice-related findings. The HCI indicator slide reporting from the districts has improved over the last few months, but not all districts are doing it. Over the next few months, with regular review and refresher trainings planned, we expect all the districts to start reporting regularly. This is expected to further increase the achievements in the above indicators.

We noted a higher proportion of people with current facility at primary care level in the later cohort. Among the HCIs done, there was only a marginal difference in the proportion of those who had a primary care facility as their current facility. This decentralisation is recommended by the programme guidelines,^11^ but the implementation gap remains. Facility- and health workers–related gaps in HCI have been highlighted previously that may have impacted decentralisation.^14,15^ Additionally, higher level of absenteeism at the primary care level,^16^ gaps in knowledge and skills, and a lack of assessment by the primary care facility and staff to deliver TB-related services compared to other services delivered at the primary care level have also been highlighted.^17,18^ We plan to address the knowledge and skills gap among primary care staff going forward by co-developing a standardised training and assessment material and implementing it in the state.

Low TB detection among household contacts in programme settings similar to our study has been flagged previously.^12,14,19^ Symptom-based screening of contacts may have led to missed TB detection.^20^ Gaps in availability of diagnostics like X-rays and rapid molecular tests at the primary care level may be contributing to this.^21^ Additionally, microscopy available at the primary care level was hardly used for bacteriological confirmation compared to NAAT-based tests at the secondary or tertiary care level during the second cohort. While NAAT-based tests have higher sensitivity to detect TB,^22^ increasing focus to improve proportion of NAAT-based tests for bacteriological confirmation in programme settings^10^ may have led to overall lower notification. While we wait for access to NAAT in primary-level facilities,^23^ sputum microscopy should not be done away with in these facilities. This might have contributed to the slight improvements in HCI not translating into improvements in TB diagnosis among household contacts (overall and among children [<5 years]). Individual-level data of all household contacts, those diagnosed with TB and initiated on TPT, are not available. This may have contributed to lower diagnosis. Ideally all contacts need to be entered in *Ni-kshay* as either presumptive TB or TPT beneficiary. There are the gaps in recording of data reported previously.^24^ Availability of individual-level data will help programme managers to track those individuals whose diagnosis or TPT initiation is pending and act on it. Proportion of TPT initiation overall, even though higher in October–December 2025, is below the programme expectation of 90%.^3^ Efforts to bridge this gap as part of our implementation work will be continued.

Drop in TPT initiation among children (<5 years) is cause of concern. TPT initiation among those < 5 years has long been part of the programme, prior to its recent expansion to other age groups. Apart from further dilution of focus by expansion to other age groups, availability of TPT, acceptability by parents of the need for medicines in asymptomatic children, and lack of knowledge among primary care staff could have been major contributors.^25–27^

Early implementation findings have key lessons for policy and practice change. There is a need for improving access to diagnostics (NAAT, microscopy, X-rays) for household contacts to detect more people with TB. Individual-level data for those initiated on TPT need to be entered and monitored in the programme. Decentralisation to primary care will be helped by these two measures. Children <5 years need to be prioritised for TB diagnosis and TPT implementation. Challenges in TB diagnosis at primary care level among children (<5 years) can be a future area of operational research. These findings will be shared with the state health department and follow-up for reinforcing districts to prepare HCI indicator slide will continue. District-wise training to address individual-level data entry gaps and focus on children(<5 years) will be done. Additional efforts to improve access to diagnostics, and access to TPT and follow-up, will be needed to reduce TB incidence.

## CONCLUSION

Early implementation changes reveal marginal improvement in HCI coverage and TPT initiation following the introduction of an HCI monitoring indicator slide at the district and state level in Chhattisgarh NTEP. This change may not be attributable to the implementation only. Gaps in access to diagnostics and HCI for children remain. There is a need for individual-level data on all household contacts identified by the programme to further strengthen this component. Future implementation will focus on these identified priorities and HCI investigation slide-based monitoring for all districts in the state to further improve TB care in the state.
